# Pathological Diagnosis

**Published:** 1878-02

**Authors:** 


*A Compend of Diagnosis in Pathological Anatomy; with Directions for making Post Mortem Examinations.* By Dr. Johannes Orth, First Assistant in Anatomy at the Pathological Institute at Berlin. Translated by F. C. SHATTUCK, M. D., and G. K. SABINE, M. D. Revised by R. H. FITZ, M. D., Assistant Professor of Pathological Anatomy in Harvard University. New York: Hurd & Houghton. Buffalo: Peter Paul & Bro.

Ths author is an assistant to Professor Virchow to whom with Professor Rindfleisch, a former teacher, he dedicates his book, which we may expect does justice to the views of these most distinguished authorities in pathology. Several years experience as a teacher in pathological work and opportunities for investigation which are thought to be unsurpassed, has enabled the author to present the subject in a most desirable way for those who are liable to the ordinary difficulties of a beginner in practical pathology and at the same time with very creditable thoroughness, completeness and freshness. There is a clearness and intelligibility in the statements of the work that convinces one who has slight acquaintance with microscopical observations, that they have been dictated by experience and that theories have been properly used to explain experimental facts. There is far more pleasure in following such a practical guide than in reading elaborate sections on pathology in general works on theory and practice of medicine, at least until the way has become pretty well known to one.

The lately promulgated Prussian Regulations for forensic physicians in making autopsies are adopted and the pathological views generally current on the continent, are there presented. A very good idea of the whole subject is presented as the tabular view of contents which occupies sixteen pages. One has a great advantage in use of this work, who has an easily acquired degree of ability in use of the microscope and can follow the plain directions for microscopical examination of parts.

The subject of cancers is clearly presented. Three divisions, scirrhus, encephaloid and colloid, are made of ordinary cancer, and epithelioma or cancroid is divided into two kinds, infiltrating and papillary. The different features of these growths in gross and microscopically, are well described.

Transitional forms between pure adenoma of the breast and cancer occur. Secondary or metastatic cancer of the liver proceeds most frequently from cancer of the stomach, also from the uterus, rectum, breast and esophagus. Metastasis from almost all recognized forms of cancer have been observed in the liver and the cells and often the interstructure present there the same characteristics as in the primary growth. Thus metastasis from cancer of the stomach, uterus, rectum and ovaries possess cylindrical cells, those from the esophagus, cervix uteri, etc., are the so-called cancroids, while those from cancer of the breast are composed of irregular cancer cells resembling glandular epithelium.

Tubercle of the lung is said to be found in three forms,—1. Disseminated uberculosis where the smaller miliary tubercles are found scattered pretty uniformly and following the course of the lymphatics and blood vessels. 2. Localized tuberculosis which in one form, frequent in childhood, occurs about a single cheesy nodule in the lungs or about the bronchial glands or again it occurs in adults in connection with cheesy pneumonia or peri bronchitis. 3. Bronchitic tuberculosis which affects the mucous lining of the bronchi. In respect to the distinction between tubercular and inflammatory products in the lung, he says it is pretty safe to assume in most cases that the effect is not purely inflammatory but of a mixed or tubercular and inflammatory character, even when the pathological changes must be ascribed in great measure to true inflammatory processes.

The sections on diseases of the kidney, liver, spleen, etc., are quite noticeable and conform mainly with statements in other quite recent works. There are very many facts of interest to be gathered from the work which is a most desirable one for students and is valuable to the profession for its new ideas and for practical service as a manual of pathological diagnosis. W.

